# Supplementary material for: Similarities between decapod and insect neuropeptidomes
Source: PeerJ. 2016 May 26;4:e2043. doi: 10.7717/peerj.2043 (PMC4888303; doi:10.7717/peerj.2043)
Supplement: Supplemental Information 2 — The number of individual reads found in different SRAs from cDNA prepared from eggs and eleven tissues of Carcinus maenas. Note that the numbers refer to the individual reads corresponding to each gene that are present in each of the twelve SRAs. These numbers are not normalized and as the preparation of cDNA libraries includes a PCR step, such numbers are not a reliable reflection of the expression level of the genes of interest. [file peerj-04-2043-s002.pdf]

Eggs	Eye	Nerve	Intestine	Ovary	Testis	Epidermis	Muscle	Heart	Hepatopancreas	Gill	Haemolymph	
17	87	1	0	24	0	2	0	0	0	0	0	ACP
6	11	1	1	0	0	1	0	0	0	2	0	ACP-GPCR
119	823	1376	2	2	0	1	0	6	2	0	0	Agatoxin-like peptide
35	138	1093	1	1	3	4	1	0	2	1	83	Allatostatin A
14	45	115	12	11	1	42	143	25	2	1	0	AstA-GPCR
85	272	387	0	165	1	8	1	2	0	2	0	Allatostatin B (= mip)
8	5	37	8	27	3	27	3	0	29	7	4	AstB-GPCR
31	41	331	246	7	0	26	0	3	3	0	0	Allatostatin C
19	1	155	2	0	1	1	0	0	0	0	0	Allatostatin CC
7	46	76	0	53	0	3	0	0	0	1	1	Allatostatin CCC
19	17	10	2	105	4	86	1	0	0	0	0	AstC-GPCR
16	12	10	2	104	4	88	1	0	0	0	0	AstC-GPCR, splice variant
14	0	550	0	16	0	7	0	0	0	0	0	Bursicon-A
10	0	444	2	0	0	0	0	0	0	0	0	Bursicon-B
202	59	98	46	31	20	49	91	51	3	65	0	Bursicon-GPCR
24	12	11	178	0	0	0	0	0	0	0	0	Calcitonin
17	10	10	112	0	0	0	0	0	0	0	0	Calcitonin common exon
2	1	5	2	0	0	0	0	0	0	0	0	Calcitonin A-specific
18	10	1	121	0	0	0	0	0	0	0	0	Calcitonin B-specific
3	8	38	0	0	0	0	0	0	0	1	0	CCHamide 1
3	12	13	1	1	0	0	1	1	0	2	17	CCHamide 2
3	3	4	8	22	3	9	0	0	0	0	0	CCHamide-GPCR-1
7	2	9	0	25	126	7	0	0	0	0	0	CCHamide-GPCR-2
2	23	34	0	112	0	20	0	0	0	0	0	CNMamide
0	5	6	0	40	0	3	0	0	0	0	0	CNMa a specific
0	0	0	0	17	0	1	0	0	0	0	0	CNMa b specific
20	153	4	0	0	0	0	0	0	0	0	0	Corazonin
26	89	208	0	0	1	0	0	106	0	0	0	CRF-like diuretic hormone
10	6	9	0	0	0	0	0	1	0	0	0	CRF-like DH-GPCR
34	31	823	0	0	0	0	0	1	0	0	0	CCAP
22	3	23	6	25	1	11	14	9	2	1	0	CCAP-GPCRa
15	3	25	5	18	0	11	15	9	2	2	0	CCAP-GPCRb
1	353	0	0	0	0	2	0	0	0	0	0	CFSH 1
1	23	1	0	1	0	0	0	0	0	0	0	CFSH 2a
0	1	0	0	2	0	1	0	0	0	0	0	CFSH 2b
83	3418	476	101	78	25	46	26	28	14	61	4	CHH 1
111	4969	663	141	125	35	59	47	33	18	80	7	CHH 1 alternative splice product
24	366	330	2757	44	49	41	4	20	45	18	2	CHH 2
43	173	735	1	2	0	0	0	4	0	0	0	DH31
11	161	28	0	0	6	1	0	0	0	0	0	Eclosion hormone 1
10	0	0	0	0	0	1	0	0	0	0	0	Eclosion hormone 2
103	48	120	1	3	5	50	0	1	0	0	0	ETH
11	30	45	0	0	0	0	0	0	0	0	0	EFLamide
15	7	10	0	0	1	10	0	0	0	0	0	EFLamide-GPCR
5	12	32	156	0	4	3	0	0	2	0	1	Elevenin
5	8	6	81	1	0	4	0	1	3	4	4	Elevenin-GPCR-1
14	75	84	36	78	57	92	67	43	28	24	25	Elevenin-GPCR-2
33	233	191	0	1	1	34	0	1	0	0	0	FMRFamide
3	19	51	2	4	2	81	41	49	3	25	0	FMRFa-GPCR

Eggs	Eye	Nerve	Intestine	Ovary	Testis	Epidermis	Muscle	Heart	Hepatopancreas	Gill	Haemolymph	
12	46	43	0	0	0	1	0	0	0	0	0	GPA2
20	48	75	2	5	0	2	0	1	1	0	0	GPB5
80	216	788	45	41	7	64	36	80	0	1065	2	GPA2/GPB5-GPCR
1	571	3	30	0	12	1	1	0	2	0	1	Hyrg
3	1	0	0	0	0	2	0	0	0	0	0	Insulin
26	29	22	16	150	13	57	4	3	2	3	0	Insulin tyrosine kinase receptor
11	71	141	0	2	0	0	0	0	1	0	0	Leucokinin-a
11	120	228	0	0	0	0	0	0	1	0	0	Leucokinin-b
2	8	16	1	9	0	3	0	0	0	0	0	Leucokinin-GPCR
4	189	0	0	0	0	3	0	0	0	0	0	MIH
26	149	298	1	1	1	1	0	0	0	0	0	Myosuppressin
39	13	45	133	18	0	7	6	2	0	4	0	Myosuppressin-GPCR ?
28	87	81	0	0	0	5	0	0	0	0	0	Natalisin
130	952	4536	635	324	491	365	331	1444	161	1935	0	Neuroparsin 1
73	182	478	13	22	0	6	4	8	0	6	0	Neuroparsin 2
2	83	160	94	52	6	5	22	12	0	81	0	Neuroparsin 3
308	94	877	108	402	124	665	2910	986	928	727	5	Venus kinase receptor 1
357	258	1211	233	233	115	257	1119	519	131	199	17	Venus kinase receptor 2
5	28	15	0	0	0	2	1	2	0	1	1	Neuropeptide F 1a
3	22	12	0	0	0	2	1	2	0	1	1	Neuropeptide F 1b
1	1	5	0	0	0	0	0	0	0	0	0	NPF 1b specific
20	62	45	0	1	3	0	0	0	0	3	0	Neuropeptide F 2
50	603	449	8	0	0	10	0	0	0	0	1	Neuropeptide-like precursor 1
40	223	661	26	0	0	2	0	0	1	0	0	Orcokinin-A
8	5	20	1	1	2	3	0	0	0	1	0	Periviscerokinin
112	446	2	1	2	4	5	3	3	0	4	2	PDH 1
25	101	0	0	1	1	0	0	0	0	0	0	PDH 2
26	7	3	1	0	1	1	0	0	0	0	0	PDH-GPCR-1
16	18	3	1	0	0	1	0	0	0	0	0	PDH-GPCR-2
17	91	411	0	0	1	1	0	0	0	1	0	Proctolin
6	20	107	6	13	2	19	94	43	3	15	0	Proctolin-GPCR-1
12	18	119	0	7	1	10	0	3	0	0	0	Proctolin-GPCR-2
25	70	94	0	12	0	0	0	0	0	0	0	Pyrokinin
2	2	6	0	2	0	28	0	0	0	0	0	Pyrokinin-1-GPCR-2
42	182	65	0	0	0	2	0	0	0	0	0	RPCH
93	2	103	0	0	0	0	0	0	0	0	0	RYamide
4	5	5	8	2	0	2	0	0	8	0	0	Ryamide-GPCR-1
5	8	7	9	3	0	35	0	0	146	0	0	Ryamide-GPCR-2
3	26	19	2	10	1	6	0	0	0	0	0	sNPF
5	1	20	1	0	3	79	0	0	2	0	0	sNPF-GPCR-1
1	0	7	0	0	0	6	0	1	1	0	0	sNPF-GPCR-2
44	410	395	0	0	0	1	0	0	0	1	1	SIFamide
3	6	5	4	0	6	1	0	0	0	0	0	Sulfakinin
30	296	124	246	3	0	0	2	2	0	0	0	Tachykinin
8	12	31	9	18	1	45	4	4	5	8	169	Trissin
10	36	42	0	4	1	5	0	0	0	0	2	Vasopressin
4	3	2	0	0	0	11	0	0	0	1	0	Vasopressin-GPCR
9	10	7	3	52	0	1	0	0	0	0	1	CG31096 ortholog
1	2	0	0	3	0	0	1	0	0	0	0	CG34411 ortholog
