# Supplementary material for: Similarities between decapod and insect neuropeptidomes
Source: PeerJ. 2016 May 26;4:e2043. doi: 10.7717/peerj.2043 (PMC4888303; doi:10.7717/peerj.2043)
Supplement: Figure S1 — (A) Trinity generated transcript for Eriocheir FMRFamide precursor. (B) Trinity generated transcript for Scylla orcokinin precursor (Bao et al., 2015). Note that both these contigs have long internal repeats that would be highly unlikely to occur by chance and, hence, suggests that they are artefacts. Nucleotide sequences highlighted in yellow are perfect repeats. (C) Alignment of several crab orcokinin precursors, providing additional arguments to suggest that the second Scylla orcokinin precursor contig is indeed an artefact. [file peerj-04-2043-s003.pdf]

a

Eriocheir sinensis FMRFamide Trinity transcript

```
GAGGTTCGGCCGCTCTGACGCTGAGGATTTCGGTCTGCCCGGTGGCCCCCTCGCTTTTGCTAATGGTA
TGCAGGATGAGGACTTGCTCGAGGATATCCCACTGGACGAGAAGCGCGCCGGTCACAGGAACTACCTT
CGCTTCGGTCGCGGCGGCATCAAGAACAGGTTCGGACGTGCCGGCAACCGCAACTTCATTCGCTTCGG
CCGCTCCGTTGACCGCCAACTGAAGGAGGAGAAGACCCGTGACACCACCCTGAACCCCACCGCCGCCC
CCCACTCCCCAGCCAAGACCCAGGAGTCCCACCGTTCCAAGAGGTCCGCCAGTCCCTACAACCAGATC
ATGATGCCTTCCCGCGGACCCGTCGCCTGGGGCATCGACTACCAGCCCGAGGAAGAGGAGGAGCTCGA
CGAATCCCTGGACGCCCCTGAGGTGACCAAGCGTGCCTACAACAAAAGCTTCCTGCGCTTCGGCCGCG
ATCGCAACTTCCTGAGGTTCGGCCGCTCTGACGCTGAGGATTTCGGTCTGCCCGGTGGCCCCCTCGCT
TTTGCTAATGGTATGCAGGATGAGGACTTGCTCGAGGATATCCCACTGGACGAGAAGCGCGCCGGTCA
CAGGAACTACCTTCGCTTCGGTCGCGGCGGCATCAAGAACAGGTTCGGACGTGCCGGCAACCGCAACT
TCATTCGCTTCGGCCGCTCCGTTGACCGCCAACTGAAGGAGGAGAAGACCCGTGACACCACCCTGAAC
CCCACCGCCGCCCCTACAACCAGATCATGATGCCTTCCCGCGGACCCGTCGCCTGGGGCATCGACTAC
CAGCCCGAGGAAGAGGAGGAGCTCGACGAATCCCTGGACGCCCCTGAGGTGACCAAGCGTGCCTACAA
CAAAAGCTTCCTGCGCTTCGGCCGCGATCGCAACTTCCTCAGATTTGGCAAGAGGAACGACGCCCCCA
CCGAGTTCGAGATGGAGCCCCCCACCTACCCCCGCTTCCAAAGGGCCCCCTACGTAGTGAGATTCGGT
TAAGATGGAGCAGCTCTTCGGGCCGCCCCTCTCCCTTAACCTCCCTTCCCGTCCCCTCTACCTACCTA
ACCCTCTCCCGGACCGCCAGACAGACACACACATACACACATACCATATAGACACATACTCGTACATT
CCCCTATGACTTAGATGCCTAATTGAGCACCAGACAGCCAGCCAGCCAGCCGTCGCCGAGCTCTCACC
TCCTTGTTTGAACGAAGCGGCGACCACCG
```

b

Scylla paramosain orcokinin transcript 2 (KR078364.1)

```
CAAGAGGAACTTTGATGAGATTGACCGCTCTGGCTTCGGCTTCGCTAAGAGGAACTTTGATGAGATCG
ATCGCTCTGGCTTCGGGTTCGCCAAGAGGAACTTCGATGAGATTGATCGCTCCAGCTTCGGTTTCAAC
AAGAGGAACTTTGATGAGATTGACCGCTCTGGCTTCGGCTTCGCTAAGAGGAACTTTGATGAGATCGA
TCGCTCTGGCTTCGGGTTCGCCAAGAGGAACTTCGATGAGATTGATCGCTCCAGCTTCGGTTTCAACA
AGAGGAACTTTGACGAGATCGACCGCTCCAGCTTCGGCTTCGTGAAGAGAATGCTCACCCCCCGAGAC
CTCGCCAACCTCTACAAGCGCAACTTTGACGAAATCGACCGATCTGGTTTCGGTTTCGTGCGCCGCAA
CGCTGAGTG
```

c

Allignment of crab orcokinin precursors

```
Eriocheir       MTREVICTALLLTLCVMAHAGAIKDAPSQPANQPDTGYSSDGSAAKRFDAFTTGFGHSKRN
Scylla          MTRDVFCTALLLALCVMASEGAIKDAPAHANNHPDAGYPSDGSSAKRFDAFTTGFGHSKRN
Carcinus        MTRDVFCTALLLALSVMASEAAIKDAPAHPSNHPDTGYASDGSSAKRFDAFTTGFGHSKRN
                ***:*:******:*.***  .******::  *:**:** ****:*****************

Eriocheir       FDEIDRSSFGFAKR---------------LLTPRD---LYKRNFDEIDRSGFGFVRRSAE
Scylla          FDEIDRSSFGFAKRNFDEIDRSSFGFVKRMLTPRDLANLYKRNFDEIDRSGFGFVRRNAE
Carcinus        FDEIDRSSFGFNKRNFDEIDRSSFGFVKRMLTPRDLANLYKRNFDEIDRSGFGFVRRNAE
                *********** **              .:*****   *******************.**
```
