# Supplementary figures and images for: Similarities between decapod and insect neuropeptidomes
Source: PeerJ. 2016 May 26;4:e2043. doi: 10.7717/peerj.2043 (PMC4888303; doi:10.7717/peerj.2043)

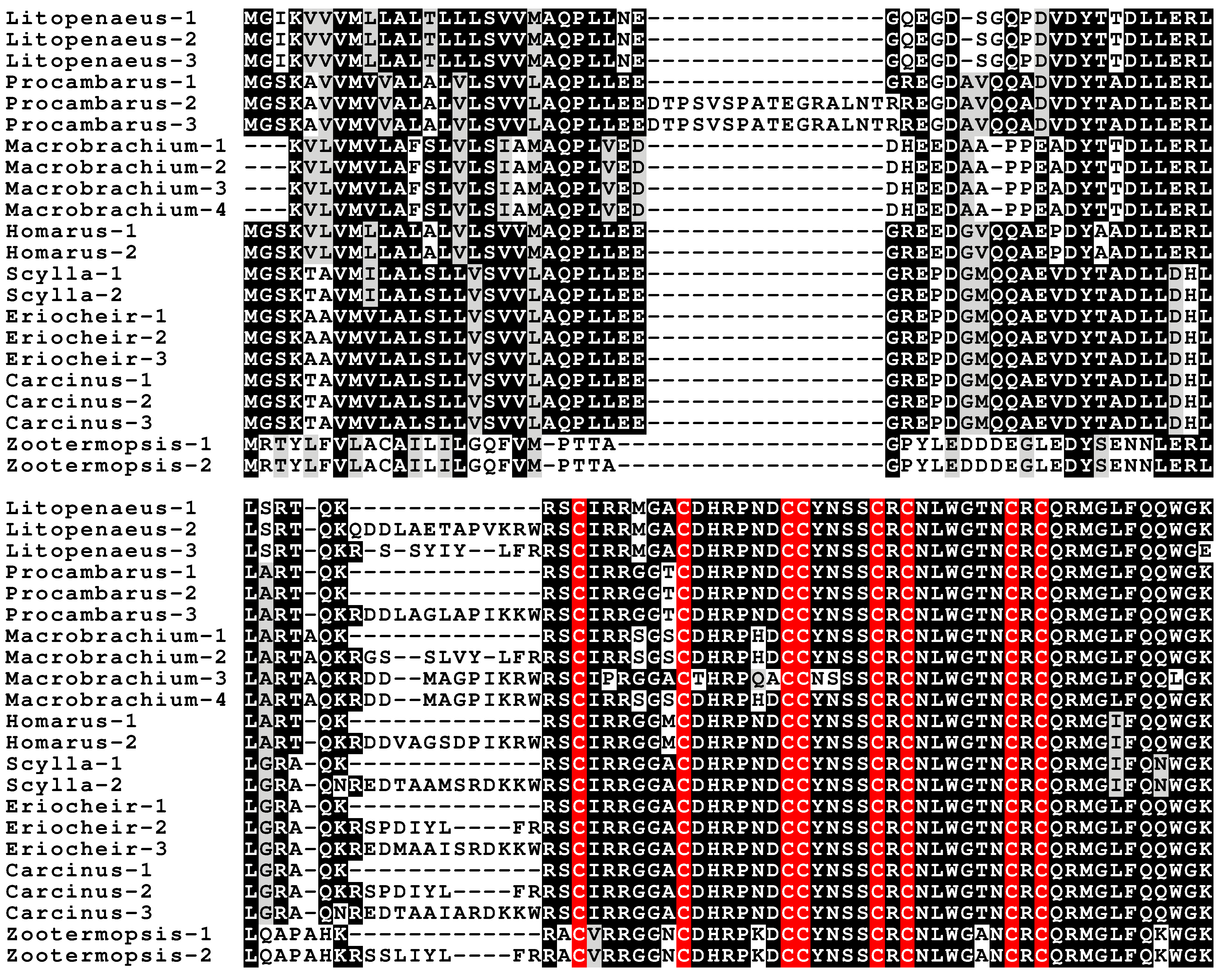

Supplement: Figure S2 — Note that both the sequence of the peptide as well as the presence of various transcripts of this gene are well conserved within decapods. [file peerj-04-2043-s004.png]

0.2

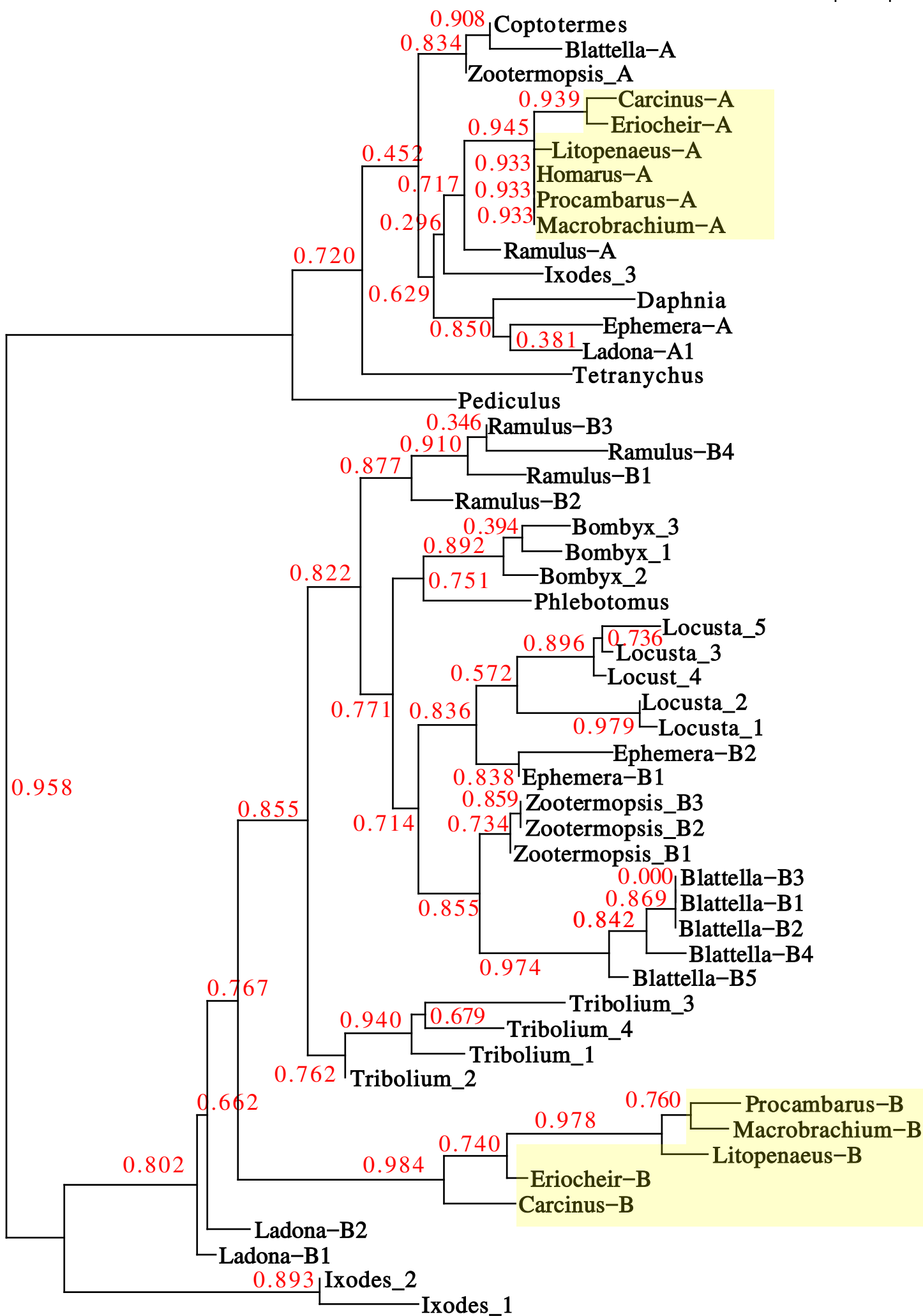

Supplement: Figure S3 — Note that the decapod calcitonins (highlighted in yellow) fit nicely in with the other arthropod calcitonins and are hence easily classified as being either of the A or B type. [file peerj-04-2043-s005.pdf]
