# Supplementary material for: Similarities between decapod and insect neuropeptidomes
Source: PeerJ. 2016 May 26;4:e2043. doi: 10.7717/peerj.2043 (PMC4888303; doi:10.7717/peerj.2043)
Supplement: Figure S4 — (A) Deduced amino acid sequence of Carcinus cryptocyanin. Note that the sequence deduced from mass spectrometry data (KIFEPLRDKN) is different from the subsequence in cryptocyanin. However, this may well be an error in sequence interpretation from the mass spectrometry data as KIFEPLRENN and KIFEPLRDKN have very similar theoretical masses (1259.41 and 1259.45 respectively, versus 1259.71 found). This does not explain the KIFEPLVA peptide sequence, but given its similarity to the other peptides, it seems plausible also related to a cryptocyanin. (B) Deduced amino acid sequence of Homarus thymosin containing the subsequence DLPKVDTALK found by mass spectrometry. (C) Deduced amino acid sequence of Homarus histone 2A containing the subsequence AVLLPKKTEKK found by mass spectrometry. The peptide sequence KPKTEKK is perhaps PKTEKK, and if so, it would also be present in histone 2A. (D) Homarus cytoplasmic type 3 actin containing the subsequence LRVAPEEHPVLL found by mass spectrometry. All protein sequences were deduced from SRAs from the respective species using Trinity. Peptides identified within these protein sequences are highlighted. [file peerj-04-2043-s006.pdf]

a

Carcinus maenas cryptocyanin

MKVLAVFGLLALSAVAAWPGYAMSDEPDGVSVHQKQHDVNYAFYKIFEPLREN
NLADKAATFNPLADFSMYKDGGDAVRHLMDELTQGHLLEKKHWAVASNKRHLE
EAIMLFEVFMQCIDWNCVASNGAYFRERVNEEEFIYAAYHAIKHSPLTQHVVL
PAMYEVKPHHFTKTQVIEEAYEAHEMKLHNVFFQTNFTGTPNDIEQRVAYFRE
DIGVGTHHLMIHLENPFWWKDTYGYHIDRKGENFFYAYHQLLNRYEAERISNH
LRPLEELKLDEPLEQGFAPQTTYKFGGPFPIRNDDIHLHDVDKLGKIHEIVHM
EDRIHDAIAHGYVEDEQGNKINIENDNGIDVLGDIIQSSMYSPNRKYYGNLTT
MAYNMLDHQTDPKNKYDTPPGVLAHLETLPRDPAAWRLHKRIDNIFREHIDSL
PPYTKDQLVFPGITVSDIQIQGNLETYFEEYKYDLVNAFNDNSSEAEFYGIYA
TMPRLNHKEFTYKINVENNNGASKKSVIRILAMPYRDGNGVIIPFDEGRWLAI
EMDLFIKTLTPGGNEITRKSSEASITVPDVPTYKTLTEMTEAHQNLEMYESAT
GIPNRLLLPKGNEEGVEFRLMVAVTDADQDVNDESIITMNKYHHYGVRGVQPD
KRPFGYPLDRRVPDEHIIDEVPNIKETMVKVYNHNVFIPIPHN-

b

Homarus americanus thymosin

MSTETHLKDLPKVDTALKGQLEAFTPDKLKKIDTEEKVTLPTKDDIETERTHQ
SIFQGVTGFDKTSMRHAETQEKIALPAKEDIVAEKGQQALRQGIEGFNPSALK
RTETQEKNKLPTKEEIEQEKKA-

c

Homarus americanus histone 2A

MSGRGKGGKVKGKSKSRSSRAGLQFPVGRIHRLLRKGNYAERVGAGAPVYLAA
VMEYLAAEVLELAGNAARDNKKTRIIPRHLQLAIRNDEELNKLLSGVTIAQGG
VLPNIQAVLLPKKTEKK-

d

Homarus americanus cytoplasmic type 3 actin gi|207298847|gb|ACI23575.1|

MCDDDVAALVVDNGSGMCKAGFAGDDAPRAVFPSTVGRPRHQGVMVGMGQKDSYVGDE
AQSKRGILTLKYPIEHGVVTNWDDMEKIWHHTFYNELRVAPEEHPVLLTEAPLNPKAN
REKMTQIMFETFNTPAMYASIQAVLSLYASGRTTGIVLDSGDGVSHTVPIYEGYALPH
AILRLDLAGRDLTDYLMTILTERGYTFTTTAEREIVRDIKEKLCYVALDFEEEMATST
QSSSLEKSYELPDGQVITIGNERFRCPEALFQPSFLGMENAGIHETTYNSIMKCDVDI
RKDLYANTVLSGGTTMYPGIADRMQKEITSLAPSTMKIKIIAPPERKYSVWIGGSILA
SLSTFQQMWISKQEYDESGPSIVHRKCF-
